# Clinical effects of topical antifungal therapy in chronic rhinosinusitis: a randomized, double-blind, placebo-controlled trial of intranasal fluconazole

**DOI:** 10.17179/excli2015-678

**Published:** 2016-02-05

**Authors:** Farshad Hashemian, Farnaz Hashemian, Najmeh Molaali, Mohammadreza Rouini, Elnaz Roohi, Saadat Torabian

**Affiliations:** 1 Department of Clinical Pharmacy, Faculty of Pharmacy, Islamic Azad University, Pharmaceutical Sciences Branch, Tehran, Iran; 2 Department of Otolaryngology, Hamadan University of Medical Sciences, Hamadan, Iran; 3 Department of Pharmaceutics, Tehran University of Medical Sciences, Tehran, Iran; 4 Department of Community Medicine, School of Medicine, Hamadan University of Medical Sciences, Hamadan, Iran

**Keywords:** chronic rhinosinusitis, antifungal therapy, fluconazole, clinical trial

## Abstract

Several studies have been in favor of fungi as a possible pathogenesis of chronic rhinosinusitis (CRS); however, to date, there is no scientific consensus about the use of antifungal agents in disease management. The aim of the present study was to investigate the efficacy of intranasal fluconazole in improving disease symptoms and objective outcomes of patients with CRS. A randomized, double-blind, placebo-controlled study was conducted on 54 patients who were diagnosed with CRS and had not been responsive to routine medical treatments. They were randomly assigned to receive either fluconazole nasal drop 0.2 % or placebo in addition to the standard regimen for a duration of 8 weeks. Patients' outcomes were evaluated according to Sino-Nasal Outcome Test 20 (SNOT-20), endoscopic scores, and Computed Tomography (CT) scores. No statistically significant difference was found in SNOT-20 (p = 0.201), endoscopic (p = 0.283), and CT scores (p = 0.212) of the patients at baseline and after 8-week course of treatment between drug and placebo group. Similar to many studies, the use of topical antifungal treatment for patients with CRS was not shown to be significantly effective. However, further studies are needed to obtain high levels of consistent evidence in order to arrive at a decision whether antifungal therapy is effective in management of CRS or not.

## Introduction

Chronic Rhinosinusitis (CRS) is defined as inflammation of paranasal sinuses, nasal cavity and mucosa which lasts more than 12 weeks and is characterized by nasal blockage, or congestion, nasal discharge (anterior/ posterior nasal drip), and/or facial pain or pressure, and/or impaired or reduced sense of smell (Fokkens et al., 2012[6]; Resenfeld et al., 2007[28]).

According to the 2011 National Health Interview Survey (NHIS), 12.8 % of adults aged 18 or over were diagnosed with sinusitis in the United States. Considering the age group, prevalence of sinusitis was highest among 65-74 year olds (16.4 %), and lowest among the age group of 18-44 (9.8 %). Comparing with prevalence of chronic bronchitis (4.4 %), hay fever (7.3 %), asthma (8.2 %), diabetes (9 %), chronic joint symptoms (29.8 %) (Schiller et al., 2012[32]), it can be concluded that sinusitis is indeed a very common condition with high disease prevalence (Hamilos, 2011[11]; Bhattacharyya et al., 2011[3]).

CRS has been found to have a negative impact on mood, energy level, physical function, and quality of life of the individuals (Gliklich and Metson, 1995[9]; Wabnitz et al., 2005[37]; Piromchai et al., 2013[24]). The impact of CRS on quality of life, measured by short form 36 scale scores (SF-36 scale scores) was found to be comparable with or worse than that of other chronic conditions such as chronic obstructive pulmonary disease (COPD), congestive heart failure (CHF), and back pain (Metson and Gliklich, 2000[22]). Moreover, it has been found that rhinitis symptoms have resulted in school absence in 26.1 % and frequent interference with daily activities in 2.7 % of the patients (Torfi et al., 2015[35]). Several factors contribute to the high economic impact of CRS. These include: being a chronic condition with no universal cure, having frequent exacerbations of symptoms which requires acute treatments in addition to the chronic ones already in place, having high impact on quality of life, having incomplete symptom control which results in seeking additional therapies to get relief, and difficulty in accurately diagnosing the condition without radiologic or diagnostic procedures (Bhattacharyya et al., 2011[3]).

Etiology of CRS is not clearly understood. Several factors have been identified to contribute to the development of CRS. These include bacterial infection, allergens, structural abnormalities such as deviated septums, biofilm bacterial infections, and Samter's triad (salicylate sensitivity, asthma, and nasal polyps) (Ryan, 2008[29]; Skadding et al., 2008[34]; Ramadan et al., 2005[27]; Palmer, 2005[23]). There is a growing body of evidence supporting the idea that fungi may indeed play a role in etiology of CRS (Ponikau et al., 1999[25]; Hashemian et al., 2012[13]; Loung and Marpel, 2005[20]; Lebowitz et al., 2002[18]; Hafidh et al., 2007[10]). However, positive fungal cultures have been found in the nasal mucosa of healthy individuals as well as patients with CRS (Lackner et al., 2005[17]). Moreover, allergic and non-allergic forms of non-invasive fungal inflammation have already been identified (Loung and Marpel, 2005[20]). Although several studies have been in favor of fungi as a possible pathogenesis of CRS, to date, there is no scientific consensus regarding the use of antifungal agents in the management of CRS (Ebbens et al., 2007[4]). One should consider that Allergic fungal rhinosinusitis (AFRS), a recognized type of rhinosinusitis (Saravanan et al., 2006[31]), is an immune-modulated disease entity and a clear immunological difference between AFRS and CRS patients has been manifested (Glass and Amedee, 2011[8]; Hutcheson et al., 2010[14]).

There is no consensus on the definitive guidelines for the treatment of CRS (Benninger et al., 2003[2]). According to the 2012 European Position Paper on Rhinosinusitis and Nasal Polyps, treatment evidence and recommendations for adults with CRS include the use of topical steroids, nasal saline irrigation, oral antibiotic therapy (less than 4 weeks during exacerbations and more than 12 weeks especially when IgE is not elevated), oral steroids, mucolytics, proton pump inhibitors (PPIs), oral and topical decongestants, probiotics, immunotherapy, and systemic and topical antifungals (Fokkens et al., 2012[6]). However, the use of a number of the above mentioned medication including systemic and topical antifungals remains controversial (Ebbens et al., 2007[4]; Sacks et al., 2012[30]).

Taking into account, the high disease prevalence, and levels of impact that CRS has on health economy and quality of life of the individuals together with its unknown etiology, and existence of no universal cure, one may recognize the urgent need for conducting well-designed clinical trials in order to arrive at high levels of evidence to improve clinical symptoms and objective outcomes of patients with CRS. Considering the emergence of the growing evidence regarding the role of fungi in etiology of CRS, to date, little randomized clinical trials (RCTs) have been done to investigate the effectiveness of topical antifungals in symptom reduction and improvement of disease outcomes. For instance, the effects of topical application of amphotericin B in patients with CRS was studied in a randomized, double-blind, placebo-controlled study. According to the results, intranasal amphotericin B had indeed reduced intranasal mucosal thickening on computed tomography (CT) scan and nasal endoscopy scores (Ponikau et al, 2005[26]). However, according to the results of another randomized placebo-controlled trial, administration of amphotericin B nasal spray to patients who underwent surgery for their nasal polyposis did not lead to significant improvements in clinical symptoms in comparison with the placebo group (Gerlinger et al., 2009[7]).

Yet, fluconazole has been found to be far less toxic than amphotericin B in numerous studies (Kontoyiannis et al., 2001[16]; Viscoli et al., 1996[36]; Abele-Horn et al., 1996[1]; Malik et al., 1997[21]). Thus, the aim of the present study was to investigate the efficacy of topical fluconazole 0.2 % in management of patients with chronic rhinosinusitis with evaluation of Sino-Nasal Outcome Test (SNOT-20), CT scores, and endoscopic scores as outcome measures.

## Materials and Methods

Fifty four patients who were diagnosed with CRS according to the American Academy of Otolaryngology-Head and Neck surgery (AAO-HNS) criteria, and had not been responsive to routine medical treatments enrolled in a randomized, double-blind, placebo-controlled study. Patients who were pregnant, lactating or suffered from a major illness (such as cardiovascular disease, acute renal or liver disease, cancer or active malignancy) were excluded from the study. The present study was approved by ethics committee of Hamadan University of Medical Sciences (Number: 16-35-9-56549) and all patients participated in the study were informed of the study procedure and signed written consent forms. Moreover, the study was registered in Iranian Clinical Trials Center (IRCT) (Registration Number: IRCT138811063186N1). The present study was conducted at Hamadan University of Medical Sciences, Besat Hospital and from 54 patients who initially entered the study, 48 were available for follow ups. Thus, 48 patients included in the study. Sample size selection was done according to a previous study of topical antifungal treatment in CRS (Ponikau et al., 2005[26]).

The patients were randomly assigned to receive either Fluticasone nasal spray 50 mcg (2 puffs per day, 2 times a day) plus fluconazole nasal drop 0.2 % (12 drops per day, 2 times a day) or Fluticasone nasal spray 50 mcg (2 puffs per day, 2 times a day) plus placebo for a duration of 8 weeks. Randomization was done by tossing a coin by an independent third party (ward secretary). Patients' outcomes were evaluated according to SNOT-20, CT, and endoscopic scores which were obtained at baseline and the end of 8 weeks period by a senior otolaryngologist. Any possible side effects were recorded. As there is no available fluconazole nasal drop in the global pharmaceutical market, it was prepared in pharmaceutics department of Tehran University of Medical Sciences. First, standard powder of fluconazole (purified fluconazole) was procured from Darou Pakhsh Pharma Chem. Co. (Tehran, Iran). Although, fluconazole is slightly soluble in water, a water-based solution of fluconazole was prepared in order to decrease possible side effects of the prepared formulation and improve patient safety in the clinical trial. No preservative was used due to possible irritating effects of a preservative on nasal mucosa. Moreover, the drug was supposed to be used in short intervals; therefore, there was probably no need for the use of preservative in the formulation. All prepared formulations were then filtered through 0.22 micron filters. Placebo was prepared in the same manner, except that distilled water was used instead of the active ingredient. In order to test the stability of the formulations, high performance liquid chromatography (HPLC) was used, and room temperature and refrigerated stability data were obtained. After a month period, 91 % of the initial active ingredient was detected. The obtained results confirmed the stability of the formulation. After completing all required tests, drug formulations were filled in 10 cc bottles obtained from SinaDarou Co. (Tehran, Iran). All procedure was done under sterile conditions. Two types of bottles were prepared, one filled with the drug formulation and the other with the same amount of distilled water. Thus, drug and placebo were exactly identical in terms of their appearance and could not be identified neither by the clinician nor the patient. Moreover, the bottles were coded by a third party who wrote down the codes in a table and the third party himself decoded the bottles at the end of the study. Data obtained from SNOT-20 scores, CT and endoscopic scores were analyzed by SPSS 18.0 software. Paired t-tests, independent sample t-tests and General Linear Model (GLM) were used to evaluate the possible differences between the drug and placebo group. P-values less than 0.05 were assumed significant.

## Results

The study included 48 of the 54 patients initially enrolled. Three patients were excluded from the treatment and 3 patients from the placebo group. One of the patients was excluded from the treatment group due to exacerbation of the disease and two others were voluntarily refused to continue the study. Three patients excluded from the placebo group were also voluntarily refused to continue the study.

No significant differences were found between the two groups regarding age, gender, smoking status and having nasal polyps before the study (P > 0.05). Demographics of the patients are shown in Table 1(Tab. 1).

Outcome measures before and after the treatment in drug and placebo groups are shown in Table 2(Tab. 2). Differences between the obtained SNOT-20 scores at baseline and the end of 8 weeks were found not to be significant between the two study groups (p > 0.05). Moreover, the obtained endoscopic scores at baseline and the end of 8 weeks treatment showed no significant difference between the two groups (p > 0.05). Differences between the obtained CT-scan scores at baseline and the end of 8 weeks were calculated not to be significant between the two groups as well (p > 0.05). 

Moreover, topical fluconazole 0.2 % was found to have a good safety profile with incidence of slight burning sensations in only 2 of the cases.

## Discussion

There is no consensus on definitive treatment guidelines for management of CRS probably due to the lack of agreement about the etiology of the disease (Benninger et al., 2003[2]). Some studies have focused on the effects of topical antifungal therapy namely amphotericin B in improving clinical symptoms and outcomes of CRS patients. According to our knowledge, to date, no one has studied the efficacy of topical (nasal drop) fluconazole treatment which is known to be far less toxic than antifungal amphotericin B in patients with CRS. Results of the present study showed no significant difference in outcome measures between the drug and placebo group at baseline and the end of 8 weeks period. In other words, no improvement was observed in SNOT-20, endoscopic, and CT scores of the patients before and after treatment. The obtained results were in accordance with the findings of some clinical trials investigating the efficacy of topical antifungal treatment in CRS.

In a prospective pilot study, application of topical nasal fluconazole spray in addition to systemic steroids and itraconazole was investigated on 16 patients with allergic fungal sinusitis (AFRS). According to the results, improvement or stabilization of disease without significant side effects was observed. Thus, the authors concluded that, topical fluconazole application may help patients with AFRS (Jen et al., 2004[15]). Nevertheless, it should be considered that allergic fungal rhinosinusitis is an immune-modulated disease entity and a clear immunological difference between AFRS and CRS patients has been manifested (Hutcheson et al., 2010[14]).

The effects of different topical nasal therapies in management of CRS have been a topic of investigation. According to a recent systematic review, there was insufficient data regarding potential benefits of topical antibiotics in patients with CRS, although topical steroids were found to be beneficial in management of CRS in patients with nasal polyps (Wei et al., 2013[38]). As thyme and honey are reported to have antibacterial and antifungal properties, the effects of thyme honey nasal spray in management of CRS was investigated in a novel randomized, double-blind, placebo-controlled clinical trial. Similar to the present study, no significant changes were observed in outcome measures between the drug and placebo group. Nevertheless, a greater reduction in endoscopic scores was observed in the treatment group (Hashemian et al., 2015[12]).

Effects of topical antifungal therapy namely amphotericin B in improving clinical symptoms and outcomes of patients with CRS have been investigated in some studies. For instance, Weschta et al. (2004[39]) studied the efficacy of intranasal amphotericin B in management of CRS in patients with nasal polyps over a course of 8-week treatment. Symptom scores were significantly worse after amphotericin B therapy; however, similar to the present study, other outcome measures were found not to differ remarkably between the drug and placebo groups. Results of another study on the efficacy of intranasal amphotericin B treatment showed significant reduction in inflammatory mucosal thickening on both CT scan and nasal endoscopy as well as decreased levels of intranasal markers for eosinophilic inflammation in the drug group in comparison with the placebo group. Nevertheless, no significant difference was reported between drug and placebo group in improving SNOT-20 scores at baseline and after treatment period (Ponikau et al., 2005[26]). The mentioned study is probably the only clinical trial that showed significant improvement in patients' outcomes after antifungal therapy. When the mentioned study is compared with similar randomized, double-blind, placebo-controlled trials of amphotericin B, one may notice that it was conducted over a longer period of time (24 weeks) and longer use of antifungal therapy may indeed have had an impact on outcomes of the patients. Moreover, one may take into account that only radiographic findings (CT and endoscopic scores) showed significant improvement and symptom reduction (SNOT-20 scores) after treatment was not significant. Results of another clinical trial on the efficacy of intranasal amphotericin B showed no significant difference in the Mean visual analogue scale (VAS) scores, nasal endoscopy scores, and Short Form-36 and Rhinosinusitis Outcome Measure-31 (RSOM-31) of the drug and placebo group (Ebbens et al., 2006[5]). However, one might consider the dosage of amphotericin B used in the mentioned study as the major weakness of the study design. Since amphotericin B at a concentration of 100 microgram/ml was found not to inhibit fungal growth in comparison with a 200 and 300 microgram/ml over a 6-week period *in vitro* (Shirazi et al., 2008[33]), a 100 microgram/ml concentration used in the mentioned study, most probably did not reach the required concentration *in vivo*. However, there is still controversy regarding the optimum dosage of antifungal treatment. According to another double-blind, placebo-controlled study, amphotericin B irrigation improved symptoms and endoscopic scores but did not show superiority to saline irrigation alone in CRS patients over a course of 4 weeks treatment (Liang et al., 2008[19]) which is consistent with the results of the present study.

Additionally, a meta-analysis was conducted on the possible therapeutic effects of antifungal therapy in CRS. All randomized, placebo-controlled trials meeting the eligibility criteria were entered the study. The results of the meta-analysis indicated that there is no evidence of beneficial effects of topical antifungal therapy in management of CRS which is in accordance with the results found in the present study. However, the authors concluded that factors such as diversity in the surgical states of the patients, concentration of antifungals and outcome measurement tools in the studies may have led to heterogeneity of treatment outcomes and should be considered as well. Thus, no definite conclusions could be made regarding antifungal therapy in CRS (Sacks et al., 2012[30]).

## Conclusion

The use of antifungal treatment for patients with CRS was not shown to be effective in the present study. However, this study was unique in the fact that most probably no one had studied the use of topical fluconazole 0.2 % in patients with CRS and the present study design allowed that the patients were not deprived of their routine treatment. Yet, further studies need to be carried out to investigate whether modifications in drug dose, route of administration, and treatment course could improve clinical symptoms and objective outcomes of the patients with CRS.

## Acknowledgements

Academic research fund was provided by Hamadan University of Medical Sciences.

## Conflict of interest

The authors declare no conflicts of interest at all.

## Figures and Tables

**Table 1:**
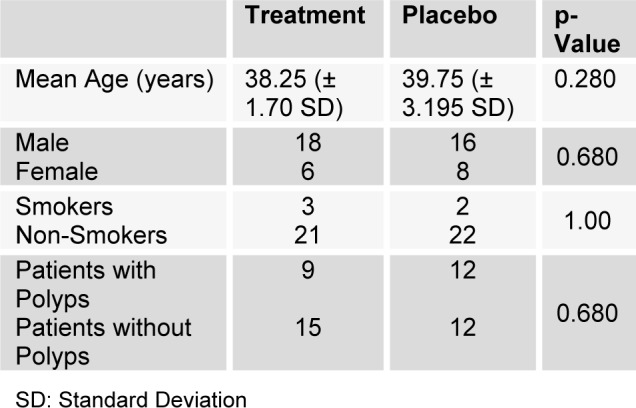
Demographics of the patients

**Table 2:**
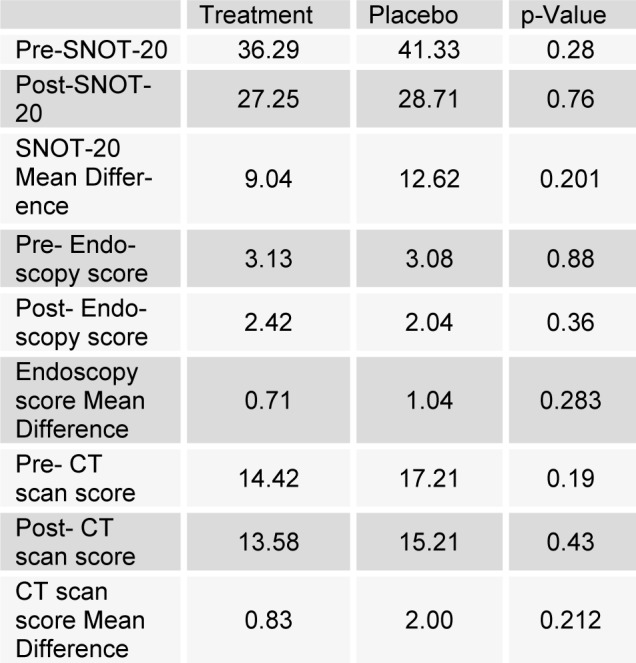
Outcome measures before and after the treatment
